# Design of All-Dielectric Metasurface-Based Subtractive Color Filter by Artificial Neural Network

**DOI:** 10.3390/ma15197008

**Published:** 2022-10-09

**Authors:** Jinhao Wang, Zichun Lin, Ye Fan, Luyao Mei, Wenqiang Deng, Jinwen Lv, Zhengji Xu

**Affiliations:** 1 School of Microelectronics Science and Technology, Sun Yat-sen University, Zhuhai 519082, China; 2 Guangdong Provincial Key Laboratory of Optoelectronic Information Processing Chips and Systems, Sun Yat-sen University, Zhuhai 519082, China

**Keywords:** subtractive color filter, structural color, metasurface, meta-atom, artificial neural network, long short-term memory, multilayer perceptron

## Abstract

Structural colors produced by light manipulating at subwavelength dimensions have been widely studied. In this work, a metasurface-based subtractive color filter (SCF) is demonstrated. The color display of the SCF is confirmed by finding the complementary color of colors filtered by SCF within the color wheel. In addition, two artificial neural network (ANN) models are utilized to accelerate the metasurface forward prediction, and the long short-term memory (LSTM) shows much better performance than traditional multilayer perceptron (MLP). Meanwhile, we train an inverse ANN model established with LSTM to recover the optimal geometric parameter combinations of the meta-atoms. With the variation of the geometric parameters of meta-atoms, versatile color displays of structural colors are realized. The metasurface we propose exhibits good performance of transmissive-type structural color in visible range. The work provides a method for high-efficiency geometric parameter prediction, and paves the way to nanostructure-based color design for display and anticounterfeiting applications.

## 1. Introduction

In the entire electromagnetic wave spectrum, only the light in a narrow wavelength band from 380 nm to 780 nm can be captured and recognized by the human eye [1]. Traditional pigment-based color displays are generated by their own chemical properties, which reflect or absorb light through specific chemical bonds. Compared with the colorant, nanotechnology-based structural colors are the results of the interaction between light and nanostructures (e.g., Mie resonances [2]), and have many superior qualities, such as being environmentally friendly and fade-resistant. Recently, excitement has been drawn stemming from the numerous potential applications of structural color, including the ultrahigh-resolution color display, sensing, holographic color display and anticounterfeiting [2,3,4,5,6].

In the past two decades, structural colors produced by light manipulating at subwavelength dimensions have been widely studied [7,8]. The colors are generated from certain optical resonances defined by the geometry and material of nanostructures, which influence the transmission or reflection of incidence beams. Since the first report of extraordinary optical transmission caused by metallic plasmonic resonance was demonstrated in 1998 [9], plasmonic structures have been considered as having a crucial role to accomplish color display and information storage applications [10,11,12]. However, the structural colors generated by dielectric structures show better performance in color saturation and International Commission on Illumination (CIE) 1931 chromaticity diagram coverage [13]. Furthermore, due to the subwavelength scale, the fabrication of such metasurface-based devices can be easily performed with the complementary metal–oxide semiconductor (CMOS) fabrication facilities, which are regularly used in the integrated circuits industries [14,15,16,17]. Therefore, these metasurface-based color filters are enabling large-scale and commercial production.

Previous reports mainly focus on the investigation of additive color filters (ACF), which work in either transmission mode or reflection mode in a narrow wavelength band [8,18,19]. Thus, the transmittance or reflectance of ACFs are relatively low. Fortunately, the subtractive color filter (SCF) can overcome this challenging task. The principle of SCF is to filter the complementary color within the frequency spectrum to obtain the target color. Since only the light in a narrow band is filtered, the transmission or reflection efficiency of SCF is higher than ACF [20,21,22].

The traditional strategy for structural color design heavily relies on the full electromagnetic (EM) simulation, such as finite difference time domain (FDTD) or finite element method (FEM). The target optical spectrum is obtained by tuning the geometries of nanorods. However, as the complexity of the nanostructures and the scale of the metasurface increase, it may take several days or weeks to design such devices via conventional methods, which will have a large cost in time and computational power. In addition, a simple picture contains millions of pixels; it is impossible to acquire the structures corresponding to the colors in every single pixel by the EM simulation-based trial-and-error method. Thus, the conventional methods are hardly satisfying for the real color display application.

By emulating the biological neural network, the artificial neural network (ANN) can learn the hidden relationships between input and output data. So far, this advanced technology has made remarkable achievements from computer vision to natural language processing [23]. In recent years, ANN has been introduced in micro–nano photonics device design, integration and measurement [24]. In the field of optical device design or optimization, ANN builds hidden relationships between the geometry of nanostructures and optical resonance. As an efficient approach enables one to deal with the tasks in the field of micro–nano photonics, numerous achievements in ANN-based optical response prediction have been proposed, including the transmission/reflection spectra [25,26] and phase response [27,28,29] prediction. Furthermore, by directly constructing the hidden relationships between color values (x, y, Y) and geometry of meta-atoms, the precise forward and inverse predictions of Si-structural colors were demonstrated in 2019 [13]. However, previous work only realized the ACF based Si-structural color design by ANN, which inevitably caused the low utilization of the light.

The multilayer perceptron (MLP) is an ordinary type of ANN that has been widely used in many scientific fields such as modeling ultrasonic welding of polymers [30] and the prediction of the efficiency of distilled water yield [31]. Previous reports about metasurface design with ANN commonly used MLP [13,29,32,33]. Currently, the recurrent neural network (RNN) has been widely used to solve the problems of sequential data. The long short-term memory (LSTM) neural network is an advanced form of RNN. Such a neural network has been widely used to solve the problems of long sequential data, such as continuous speech signals. Compared with ordinary RNN, the hidden unit of LSTM contains three gates, which are named the forgetting gate, input gate and output gate. The three gates determine and update the cell states continuously, which help the LSTM to memorize the information more remotely [34]. In current research, LSTM is not only used in natural language processing (NLP) but is also added in other fields, such as the laser cutting performance prediction [35], the prediction of water yield by solar still [36] and nanophotonics device design [37]. To the best of our knowledge, the metasurface-based SCFs designed by LSTM has not been studied so far.

In this work, we demonstrate SCFs based on a silicon-on-insulator system. A larger coverage area of the CIE 1931 chromaticity diagram than the previous report is illustrated in the field of SCFs, especially for the color green [38,39]. In addition, in order to further improve the accuracy and efficiency of the metasurface design, both the LSTM and MLP model are used to accelerate the metasurface forward design. The results indicate that the performance of LSTM for predicting the optical response of metasurface is much better than MLP. Meanwhile, the metasurface inverse design is realized. We use the tandem network architecture as the inverse ANN model [32], and the LSTM is chosen to build the inverse neural network. The inverse model can successfully generate the corresponding geometric parameters of thousands of target colors. The results indicate that ANN can be applied as a very efficient tool in simplifying the design of nanophotonics devices. Our method will pave the way to metasurface-based structural color design, and will greatly promote the development of advanced applications such as color display and anticounterfeiting.

## 2. Results

**Figure 1:**
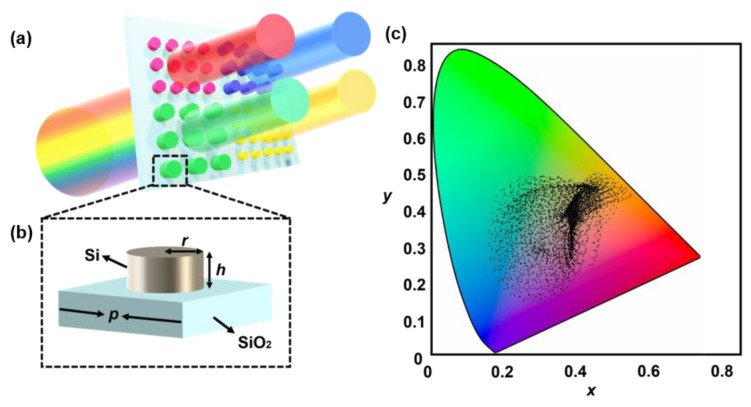
(**a**) Schematic of the SCFs illustrating the color filter effect. (**b**) Single nanopillar as metasurface unit cell of the SCF. (**c**) The coverage of generated colors by SCF on the CIE 1931 chromatic diagram.

The schematic of the metasurface-based transmission-mode SCFs is illustrated in Figure 1a. We chose the Si cylindrical as the meta-atom to modulate the incident beam, and the SiO_2_ was chosen as the substrate of SCFs. As is shown in Figure 1b, the single-unit cell of the metasurface that we mainly investigated has three geometric parameters, including the radius (r), height (h) of each nanorod and the pitch (p) of the meta-atom. Different geometric parameters produce different optical response modulations, resulting in different colors. We used the EM simulation method FDTD to obtain the transmittance spectra corresponding with 5300 groups of different geometric parameter combinations. The wavelength band of the incident light is from 380 nm to 780 nm with an interval of 2 nm. The color values (x, y, Y) are obtained by converting the transmittance spectra through the CIE 1931 color-matching functions, while x and y define the position of colors on the chromatic diagram, and Y indicates the luminance. All the results calculated were plotted in the CIE xyY chromaticity diagram, which is shown in Figure 1c. Compared with the SCFs proposed previously, we achieved a larger coverage area of the CIE 1931 chromaticity diagram.

**Figure 2:**
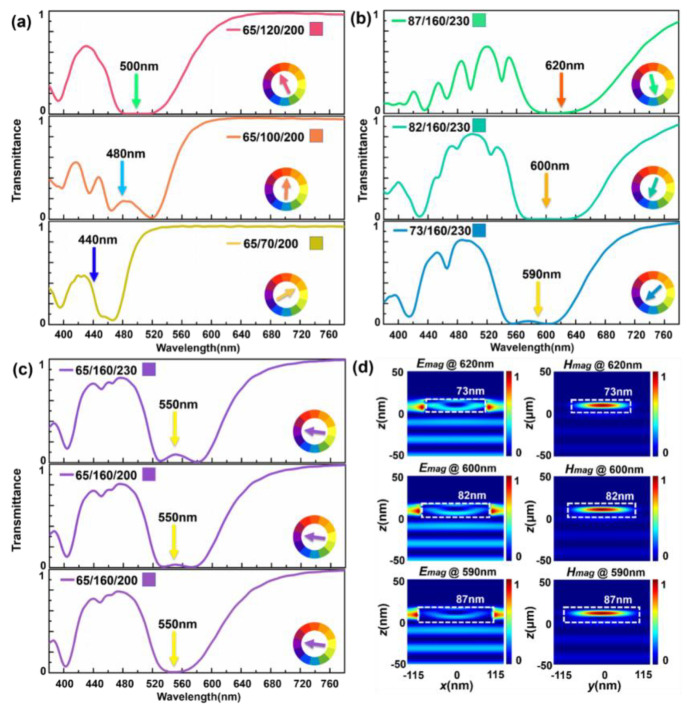
(**a**–**c**) The calculated reflection spectra of Si metasurface with different lattice sizes. (**d**) The electromagnetic field distributions of electric dipole mode and magnetic dipole mode of (**b**) at resonance wavelengths of 620 nm, (**b**) 600 nm and (**c**) 590 nm for a-Si nanopillars with radii of 87, 82 and 73 nm, respectively.

Figure 2a–c show the generated colors and their corresponding transmission spectra influenced by the geometry of meta-atoms. It clearly shows that the wavelength where the resonance occurs shifts with the change of geometric parameters, and the color calculated are thus different. The light at the resonance wavelength is filtered by SCF. Converting the spectra to (x, y, Y), the complementary color of the color defined by resonance wavelength is displayed on the chromaticity diagram. We kept the other two geometric parameters fixed, and only changed one parameter to induce the optical spectra. When r = 65 nm and p = 200 nm, h reduces from 120 nm to 70 nm, the resonance wavelength shows a significant blue shift. Filtering out the specific colors of resonance wavelengths, their complementary colors can be obtained from the corresponding position in the color wheel. The calculated colors are red (x = 0.4328, y = 0.2433, Y = 18.4622), orange (x = 0.4763, y = 0.3599, Y = 30.8985) and deep yellow (x = 0.4141, y = 0.4504, Y = 48.3004). Obviously, all the calculated results are same as the colors obtained from color wheel, which are shown in Figure 2a. In Figure 2b, two geometric parameters are fixed as h = 160 nm and p = 230 nm. As the r decreases from 87 nm to 73 nm, the resonance wavelength shows blue shift. Filtering out the specific colors of resonance wavelengths, the colors we obtain are green (x = 0.1981, y = 0.4569, Y = 15.1166), cyan (x = 0.1506, y = 0.3708, Y = 30.8985) and blue (x = 0.1734, y = 0.2184, Y = 31.0732), which are the same as the colors obtained from the color wheel. In the case of changing the p from 230 nm to 200 nm and keeping the r and h fixed, the transmittance spectrum shows few feature changes and all their resonance wavelengths are same to each other. Purple is obtained by filtering the light at 550 nm. The three results in Figure 2c are (x = 0.2688, y = 0.1628, Y = 38.0617), (x = 0.2706, y = 0.1610, Y = 37.1576) and (x = 0.2889, y = 0.1786, Y = 36.2713). The maximum color value changes calculated are Δx = 0.0201, Δy = 0.0176, ΔY = 1.7904. It is hard to distinguish the color changes by human eyes shown in Figure 2c. All the results above indicate that r and h play a more crucial role in tuning the optical resonance. Figure 2d shows the cross-section diagrams of normalized electric and magnetic fields corresponding with the structures at the resonance wavelength in Figure 2b. The edges of the nanocylinder are shown in the white dashed line. It shows clearly that a strong circular electric field is formed at the resonance wavelength and leads to the magnetic dipole enhancement within the nanopillars. Therefore, low transmittance is created and contributes to the subtractive filtering at the target wavelength. The electric and magnetic field distributions are simulated by FDTD.

**Figure 3:**
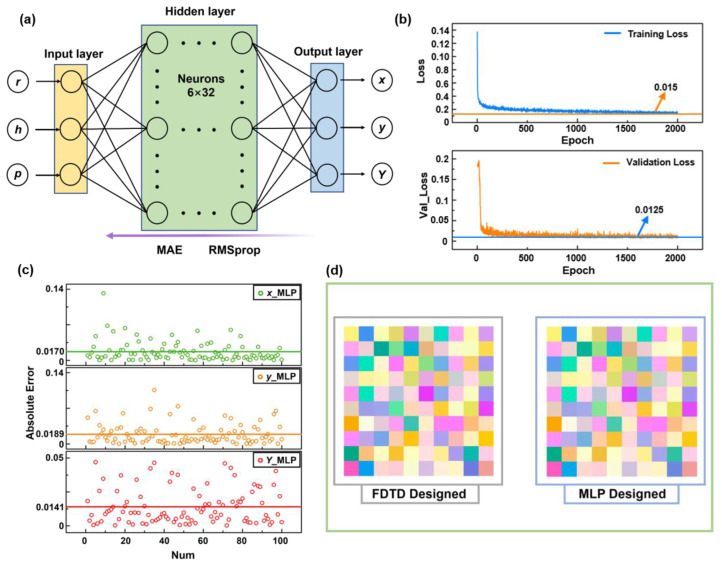
(**a**) Schematic of the MLP for predicting Si structural colors. (**b**) The loss function of training loss and validation loss in the MLP training process. (**c**) The absolute error between 100 groups of calculated color values and MLP predicted color values. (**d**) Comparison between 100 groups of real color and predicted color display.

Taking the variation of all the three geometric parameters into account, the complexity of the metasurface design will inevitably increase. It becomes difficult to obtain the geometric parameters for the target optical response by using the traditional EM simulation. In recent years, ANN has been introduced into the field of micro–nano photonic device design, which is supposed to solve the problem of time and computational power consumption. At the very beginning, we trained the forward ANN to predict the optical response corresponding to the specific combination of geometric parameters. We started with a traditional MLP network as the forward model for predicting the Si colors accurately and instantly. TensorFlow and Keras were used to establish the basic architecture of MLP. Figure 3a illustrates the structure of the forward MLP model. The optimum MLP network contains six hidden layers; each layer is constructed from 32 neurons. The activation functions used in all the hidden layers are LeakyReLU, and sigmoid is used in the input layer and output layer. Moreover, MAE is selected as the loss function. Adam is used as the optimizer to realize the backpropagation algorithm. We input the geometric parameters (r, h, p) into the ANN and output the color values (x, y, Y). All 5300 data shown in Figure 1c were used as the dataset to train the network. During the training process, 5% of the dataset were separated as the validation data and 100 groups as the test data. All the rest of the data were employed as the training data to update the weights of ANN. The complete training process contains 2000 iterations (epochs = 2000). The batch size is 256 and the learning rate is 0.001.

Figure 3b shows the changes of training loss and the validation loss. The error values decline continuously as the epochs increase. The training error drops to 0.015 and the validation error becomes about 0.0125 after 1500 epochs of training. In the subsequent training, the loss value barely decreases, indicating that the training is complete. In order to verify the efficiency and accuracy of our forward MLP neural network, 100 groups of data are used for testing. The results predicted by MLP are compared with the colors obtained by EM-simulated spectrums, and the absolute error is calculated. As is shown in Figure 3c, the top, middle and bottom subgraphs represent the absolute errors of x, y and Y values, respectively. The mean absolute errors are 0.0170, 0.0189 and 0.0141, respectively. It proves the high accurate prediction of our MLP model according to the given geometric parameters. The prediction time cost of MLP is about 24 ms, and the EM-simulation software FDTD takes more than 1 min to calculate the optical spectra. Figure 3d illustrates the comparison between 100 groups of test data and predicted colors. Both results are almost the same.

Although MLP shows good performance for color prediction with the given geometric parameters, a more accurate color design is still in high demand. It can be seen in Figure 2 that the continuous changes of a single geometric parameter cause the resonance wavelength shift, which indicates that the color values (x, y, Y) also change regularly. Compared with the MLP network, RNN is more suitable to handle the problems of sequential data. In 2019, RNN was used to accomplish the prediction of an absorption spectrum generated by random silver micro–nano structures [40]. LSTM is a special type of RNN. Compared with ordinary RNN, LSTM has better performance in dealing with long sequential data.

**Figure 4:**
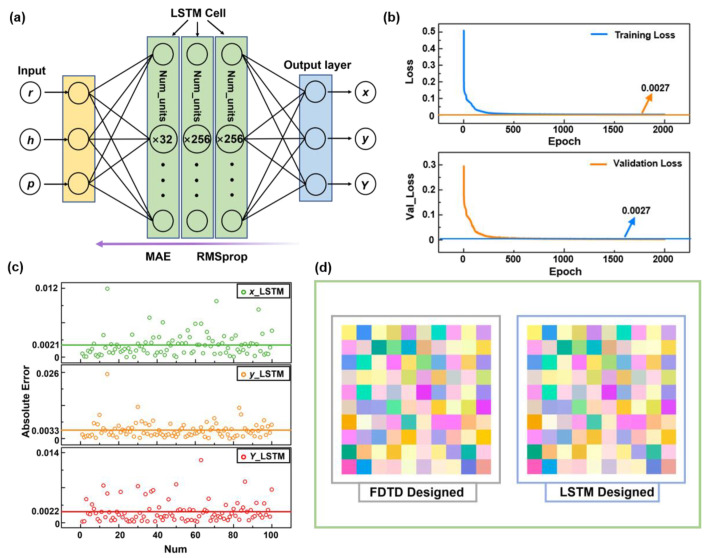
(**a**) Schematic of the LSTM for predicting Si structural colors. (**b**) The loss function of training loss and validation loss in the LSTM training process. (**c**) The absolute error between 100 groups of calculated color values and LSTM predicted color values. (**d**) Comparison between 100 groups of real color and predicted color display.

Similarly, we input the geometric parameters (r, h, p) into the LSTM model and output the color values (x, y, Y). Figure 4a shows the schematic of our LSTM neural network. Tensorflow and Keras were used to establish the basic architecture of LSTM. The optimum LSTM network contains three hidden layers with 32, 256 and 256 neurons, MAE is reused as the loss function and the optimization algorithm is Adam. All the data employed to train the MLP model were used again in this task. Similarly, 5% of the dataset were separated as the validation data and 100 groups as the test data. The complete training process contains 2000 iterations (epochs = 2000). The batch-size is 256 and the learning rate is 0.001.

Figure 4b shows the changes of training error and the validation error during the training process. The training error values decline continuously to 0.0027, and the validation error decreases to a same constant after 500 epochs. During the subsequent training, the loss value barely decreases, indicating that the training is complete. Obviously, the decreasing rate of the loss value in LSTM training is much faster than that in MLP, and the oscillation of the loss value change in the training process can be neglected. In addition, with the same number of training epochs, the loss value of LSTM is much lower than that of MLP, indicating that LSTM converges easier than MLP. To further verify the efficiency and accuracy of our forward LSTM neural network, 100 groups of data were used for testing. The results predicted by LSTM were compared with the colors obtained by EM simulated spectrums, and the absolute error was calculated. As is shown in Figure 3c, the top, middle and bottom subgraphs represent the absolute errors of x, y and Y values, respectively. The mean absolute errors are 0.0021, 0.0033 and 0.0022, respectively. It proves that LSTM shows much more accurate predicting performance than MLP. The prediction time cost of LSTM is about 26 ms, which differs little from MLP. Figure 4d illustrates the comparison between 100 groups of test data and predicted colors, and it is hard to distinguish the difference by human eye. All the results demonstrated in Figure 4 indicate that LSTM is more efficient and powerful in metasurface design.

Utilizing ANN, the accurate forward metasurface design is achieved. The well-trained ANN enables the EM simulations to be replaced to accelerate the optical response prediction, which greatly reduces the time and computational power cost. However, the forward neural network can only accelerate the forward metasurface design, and a large amount of trial and error is still needed to obtain the appropriate geometric parameter combination. In the real applications, one pattern may contain tens of thousands of pixels, and the color of each single pixel might differ from others. In order to further reduce the design time, ANN is expected to realize the metasurface inverse design. The inverse ANN outputs the corresponding geometric parameters directly by taking the target values (x, y, Y) as the input data.

**Figure 5:**
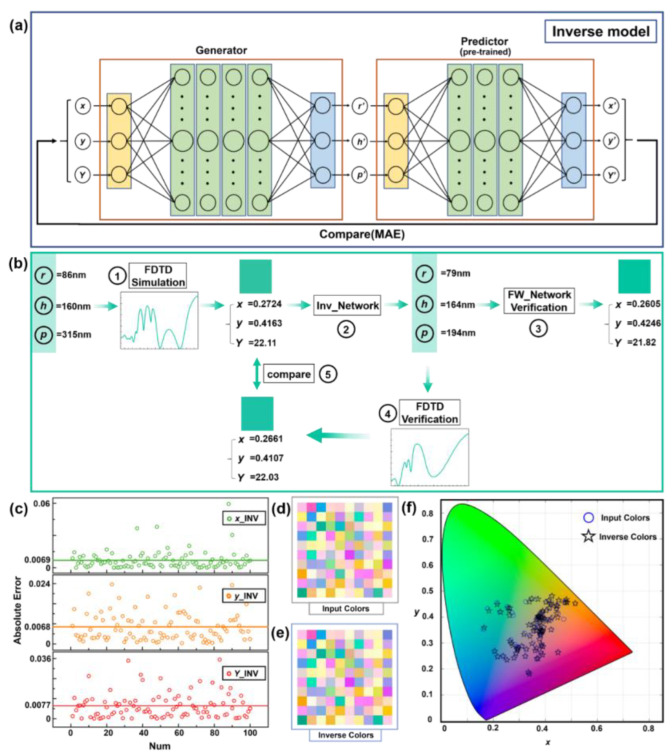
(**a**) Schematic of inverse ANN for predicting the micro–nano structures. (**b**) Metasurface inverse design and the results’ verification process. (**c**) The absolute error between the simulated colors and inverse-designed structural colors. (**d**,**e**) The 100 groups’ colors designed by FDTD and inverse ANN. (**f**) The coverage of 100 groups’ real and inverse colors by SCF on the CIE 1931 chromatic diagram.

The inverse design is more complicated than the forward design. Directly taking the colors as the input data and the geometric parameters as the output data will cause nonconvergence of the ANN. Different combinations of geometric parameters may produce the same (x, y, Y) values, which is known as the non-uniqueness problem. To deal with this challenging task, we use the LSTM to construct a tandem model to accomplish the inverse design of metasurface-based structural colors. The architecture of our inverse neural network is proposed in Figure 5a. We start by using a well-trained forward LSTM neural network as the predictor. Then, an inverse network named generator is established, which takes the colors (x, y, Y) as the input and outputs the geometry of meta-atoms (r’, h’, p’). We input the (r’, h’, p’) into the predictor to forecast the corresponding results (x’, y’, Y’). Throughout the whole training process, the weight of the predictor is fixed, and the weight of the generator is trained constantly to minimize the differences between (x’, y’, Y’) and (x, y, Y). In this part, the LSTM model is used to build the generator, which contains three hidden layers with 32, 256 and 256 units, respectively. The dropout of each hidden layer is 0.2. The output layer of the generator is a simple full-connection layer, and the activation function of it is sigmoid. MAE is used as the loss function.

We take an example to demonstrate the superior performance of the inverse ANN; the details are shown in Figure 5b. Step “1” is the electromagnetic wave simulation process. The transmittance spectrum is obtained by inputting the specific geometric parameters into the EM simulation software. The color value generated by the spectrum is (x = 0.2724, y = 0.4163, Y = 22.11), which is calculated by the color-matching function. In step “2”, we input the color generated in step “1” as the target color into the inverse ANN, and the designed geometric parameters are produced. Then, we use the well-trained forward ANN to predict the color of the designed geometric parameter in step “3”. The output color values are (x = 0.2605, y = 0.4246, Y = 21.82). The error between two groups of color values is negligible, which preliminarily indicates that our inverse ANN can successfully design the corresponding micro–nano structure according to the target color. In order to further verify the accuracy of the inverse design, we input the designed geometric parameters into the EM simulation software FDTD solution for calculation in step “4”, and convert the transmittance spectrum to the color values (x = 0.2661, y = 0.4107, Y = 22.03). In step “5”, we compare the designed color with the target color, and it is hard to observe the difference by human eye. Furthermore, the geometric parameters inputted in step “1” and step “2” are completely different, but the colors produced are almost the same, which proves the existence of the non-uniqueness problem.

To exhibit the universality of our inverse model, 100 groups of colors are selected randomly as the test data to realize the Si-structure inverse design. Figure 5c shows the absolute errors between the target value (x, y, Y) and the designed value (x’, y’, Y’). The top, middle and bottom subgraphs represent the absolute errors of x, y and Y, respectively. The mean absolute errors are 0.0069, 0.0068 and 0.0077, respectively, Figure 5d,e shows the comparison between 100 groups of target and designed colors, and we plot both data into the CIE 1931 chromatic diagram; all the results illustrated prove that the inverse design is highly precise.

**Figure 6:**
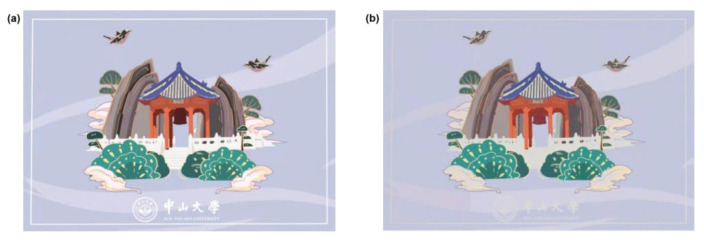
(**a**) The desired colors of the painting “Sun Yat-sen university-Xing Pavilion”. (**b**) The inverse-designed structural colors by ANN.

Finally, to demonstrate the superiority of our inverse design approach in real applications, we chose a painting, “Sun Yat-sen University-Xing Pavilion” by Lin Xiwang and Lan Tian from Sun Yat-sen University. The colors of all of the 500 × 354 pixels on the picture are extracted and inputted into the inverse ANN as the target colors, which are shown in Figure 6a. The corresponding geometry is produced by ANN according to the target colors, and we input these obtained geometric structures into a pretrained forward ANN for prediction. The designed painting colors are shown in Figure 6b. Our inverse ANN shows an excellent performance for extremely accurate silicon color inverse design. However, there are a few shortcomings that can be observed. If the target colors of some pixels are too dark, the structures’ inverse design is not accurate, because it is difficult to realize the color with an extremely small Y value by Si-structures. On the other hand, some pixels with an extremely high Y value are also difficult to be designed in reverse by Si-nanorods.

Generally, our method described above enables the design of the corresponding geometric parameters by absorbing tens of thousands of target colors in an exceedingly short time. In practice, according to the designed parameters, versatile metasurface-based structural color devices can be fabricated by CMOS technology, which greatly promotes the development of anticounterfeiting, color displays and other applications. At present, the precision of advanced lithography technology can reach 5 nm or even 3 nm, which means that more and more structural colors can be realized.

## 3. Conclusions

In this work, we designed a metasurface-based SCF, which achieves a larger coverage of the CIE 1931 chromatic diagram than previous studies. In addition, by using the MLP and LSTM neural networks, the acceleration of the metasurface forward design is realized, which greatly reduces the computational power and time cost of the metasurface design. The results prove that the performance of LSTM is much better than that of the MLP network, which also shows that ANN has the potential to replace the traditional EM simulation method. More importantly, we construct an inverse neural network model, which outputs the corresponding geometric parameters by inputting the target color. The inverse ANN enables the inverse design for tens of thousands of different colors to be realized simultaneously. This work will greatly promote the development of anticounterfeiting, color display, sensing and other applications based on structural color, and paves the way for the practical application of silicon color inverse design based on ANN. A new idea has been provided for the research of the rapid and accurate design of micro–nano photonic devices. With help of the CMOS-compatible process, the devices in real applications of structural color should be investigated and fabricated in the future.

## Data Availability

The data presented in this study are available on request from the corresponding author.
